# Respiratory syncytial virus- and human metapneumovirus-associated emergency department and hospital burden in adults

**DOI:** 10.1111/irv.12234

**Published:** 2014-02-07

**Authors:** Kyle Widmer, Marie R Griffin, Yuwei Zhu, John V Williams, H Keipp Talbot

**Affiliations:** a Department of Internal Medicine, Tulane University School of MedicineNew Orleans, LA, USA; b Southeast Louisiana Veterans Health Care SystemNew Orleans, LA, USA; c Department of Medicine, Vanderbilt University Medical CenterNashville, TN, USA; d Department of Preventive Medicine, Vanderbilt University Medical CenterNashville, TN, USA; e Mid-South Geriatric Research Education and Clinical Center and Clinical Research Center of Excellence, VA TN Valley Health Care SystemNashville, TN, USA; f Department of Biostatistics, Vanderbilt University Medical CenterNashville, TN, USA; g Department of Pediatrics, Vanderbilt University Medical CenterNashville, TN, USA; h Department of Pathology, Microbiology and Immunology, Vanderbilt University Medical CenterNashville, TN, USA

**Keywords:** Burden of illness, hospitalizations, human metapneumovirus, older adults, respiratory syncytial virus

## Abstract

**Objective:**

Determine the burden of illness associated with respiratory syncytial virus (RSV) and human metapneumovirus (HMPV) in adults, especially young adults.

**Design:**

Prospective surveillance study using RT-PCR for the diagnosis of RSV and HMPV.

**Setting:**

One academic Emergency Department (ED), one academic hospital and three middle Tennessee community hospitals.

**Sample:**

We prospectively enrolled Middle Tennessee residents ≥18 years old evaluated in the emergency department (ED) or hospitalized for respiratory symptoms May 2009 through April 2010. We collected nose/throat specimens for RSV and HMPV reverse-transcriptase polymerase chain reaction (RT-PCR) testing and obtained demographic and clinical data.

**Main outcome Measures:**

Rates of ED visits and hospitalizations were calculated using the proportion of enrolled patients positive for each virus multiplied by the number of Middle Tennessee residents evaluated in EDs and/or hospitalized in Tennessee for acute respiratory illness during the study period.

**Results:**

Three thousand two hundred and fifty six patients were eligible; 1477 (45·4%) were enrolled; 1248 (84·5%) of these consented to additional testing and had adequate samples. RT-PCR identified 32 (2·6%) patients with RSV and 33 (2·6%) with HMPV. The median duration of symptoms before ED presentation was 3·3 days with RSV and 2·8 days with HMPV, and before hospital admission was 4·5 days with RSV and 3·5 days with HMPV. The annual hospitalization and ED visit rates were similar for RSV and HMPV. The hospitalization rate associated with each virus was about 10 per 10 000 persons aged ≥50 years; ED rates were approximately 2 times higher. Hospitalization rates were about 2 per 10 000 persons aged 18–49 years, with ED rates 5–6 times higher.

**Conclusion:**

RSV and MPV are associated with substantial disease in adults, with hospitalization and ED visits rates increasing with age.

## Introduction

Respiratory syncytial virus (RSV) and human metapneumovirus (HMPV) cause substantial morbidity and mortality in adults. We previously found that rates of hospitalization associated with RSV and HMPV disease in older adults were similar to those of influenza during a recent 3-year period in a population with relatively high influenza vaccination rates (72%).^1^ To our knowledge, rates for hospitalizations using laboratory confirmation of these viruses have not been determined for adults aged 18–49, nor have they been determined for emergency department (ED) visits. This study was designed to establish rates of ED visits and hospitalizations associated with RSV and HMPV infections in adults aged 18 and older using sensitive molecular techniques. Defining rates of serious illness due to RSV and HMPV may support vaccine development for the prevention of disease and drug development for treatment of RSV and HMPV infection.

## Methods

### Study design

From May 2009 through April 2010, during the novel H1N1 influenza A virus pandemic, patients ≥18 years of age with respiratory symptoms or non-localizing fever evaluated in the ED or hospitalized were enrolled prospectively into an ongoing study of influenza vaccine effectiveness.^2^ Enrollment sites included one academic ED, one academic hospital, and three community hospitals. Eligible adults included residents of Middle Tennessee, defined as Nashville (Davidson County) and the six surrounding counties (Robertson, Cheatham, Williamson, Rutherford, Wilson, Sumner). Patients were eligible if they had any respiratory symptoms (i.e., cough, nasal congestion, coryza, dyspnea, or wheezing) or non-localizing fever that had begun within 7 days prior to presentation. Consecutive eligible subjects were approached during defined ED shifts, and hospitalized patients were approached during a 24-hour surveillance period for each enrollment day, 5 days per week. ED patients who were ultimately hospitalized are included in both groups. At the time of consent, nose and throat swabs were obtained. Samples were tested for influenza and then stored for future use (if patients agreed to future use at the time of the consent).

### Demographic and clinical information

Standardized questionnaires and medical record review captured age, sex, race, medical co-morbidities, smoking (self-reported within the past 6 months), use of specific medications (home oxygen, corticosteroids, and immunosuppressants), influenza vaccination status, clinical symptoms, admission to an intensive care unit, endotracheal intubation, length of hospitalization, and status at discharge.

### Laboratory methods

Using real-time reverse-transcriptase polymerase chain reaction (RT-PCR), frozen specimens previously evaluated for influenza virus were tested for RSV using methods published by the Center for Disease Control and Prevention (CDC)^3^ and for HMPV.^4^,^5^ To insure the quality of the specimens collected, samples were tested for RNase P.

### Analyses

Subjects who gave permission for additional specimen testing beyond influenza were included in these analyses. Descriptive analyses were performed using Pearson's chi-squared test for categorical values and Kruskal–Wallis for continuous variables, using stata version 9 (College Station, TX, USA). Rates of ED visits and hospitalizations were calculated using the proportion of enrolled patients positive for each virus multiplied by the total number of Middle Tennessee residents evaluated in EDs and/or hospitalized in Tennessee for acute respiratory illness (ICD-9 codes 381–382, 460–466, 480–487, 490–493, 786, and 780·6) during the surveillance period collected from the Tennessee Hospital Discharge Data System (HDDS). The HDDS includes age, residence, and discharge date and diagnoses for each Tennessee resident discharged from a Tennessee ED or non-federal hospital. Denominators for rate calculations were age-specific population numbers from Middle Tennessee from the census annual July 2009 estimate. We calculated 95% confidence intervals (95% CI) for all rates using 1000 bootstrap samples.

## Results

### Characterization of enrolled patients

**Figure 1:**
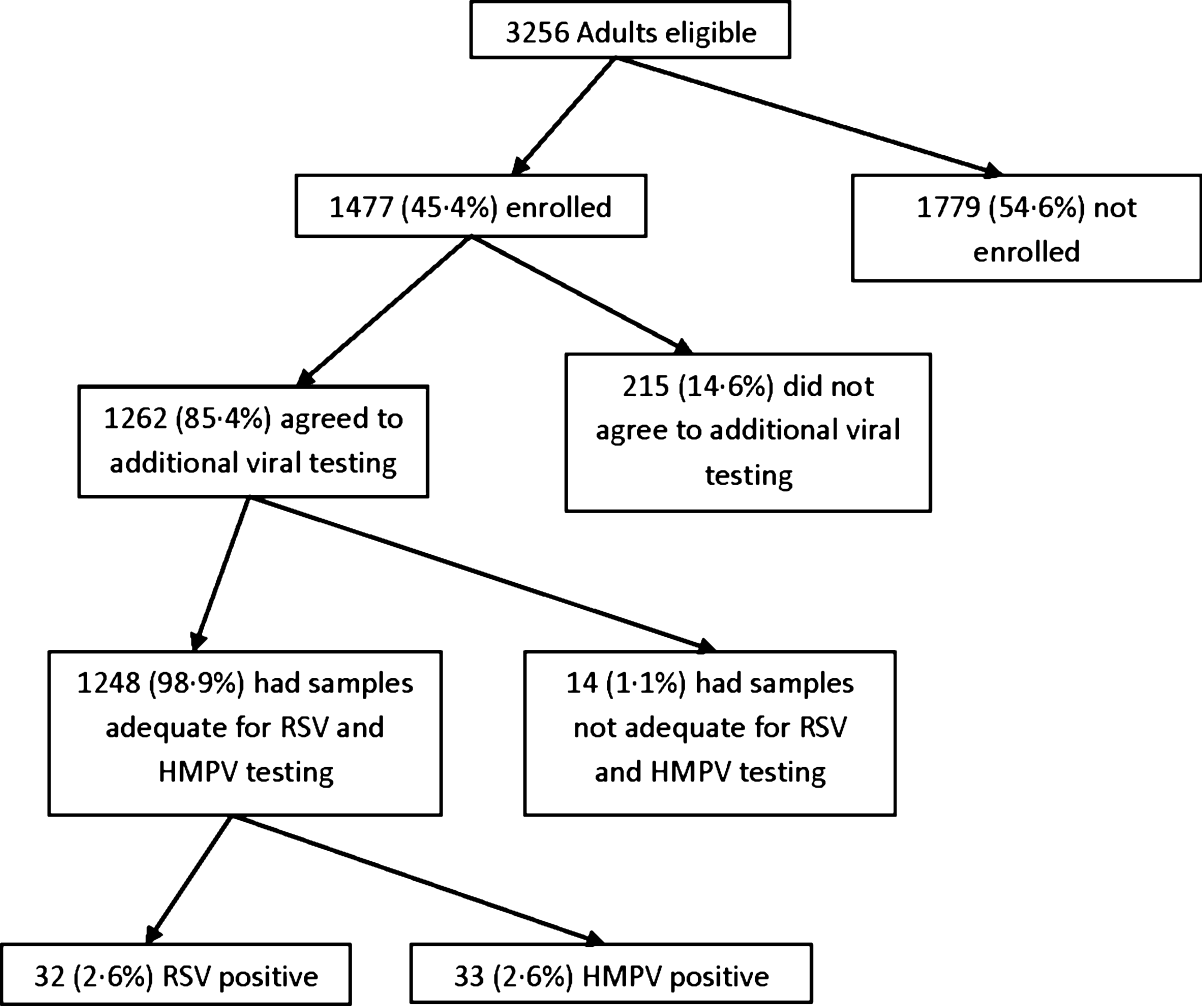
Eligibility and enrollment: 3256 were approached for enrollment, 1477 agreed to testing of specimens for influenza alone, and of those, 1262 agreed to further testing for other viruses; 1248 had adequate samples to test for RSV and HMPV.

During the 12-month study period, we identified 3256 eligible Middle Tennessee residents and enrolled 1477 (45·4%). (Figure 1) Reasons for non-enrollment included refusal by patient (61%), surrogate decision-maker (6·2%), or physician (3·6%), no legal guardian or surrogate decision-maker (6·2%), non-English language speaking (1·4%), or patient was missed or otherwise not approached prior to discharge (21·8%). Those not enrolled were older than those enrolled (median age 59 versus 53, *P* = 0·0001) and were more likely to be male (42·5% versus 38·8%, *P* = 0·018). Of those enrolled, 1262 of 1477 (85·4%) patients consented to additional viral respiratory testing beyond influenza testing for which they were originally consented. Fourteen (1·1%) of these samples had inadequate volume remaining for RSV and HMPV testing, leaving a total of 1248 patients. The 215 subjects who refused further sample testing were older (median age 63·3 versus 51, *P* = 0·0001), more likely to be male (58·9% versus 49·8%, *P* = 0·013), and Caucasian (69·8% versus 66·1%, *P* < 0·0001). Subjects whose samples were tested for RSV and HMPV had a median age of 51 years; most lived alone or with family (95·5%) and had at least one chronic illness (81·2%). (Table 1) Among those 18–49 years, 64% had a co-morbid illness versus 91% of those ≥50 years. Of the patients seen in the ED, 42% were admitted to the hospital, which was 29% of patients <50 years of age compared with 64% of those ≥50 years of age.

**Table 1:** Characteristics of patients enrolled and tested for respiratory viruses and subgroups positive for respiratory syncytial virus (RSV) and human metapneumovirus (HMPV)

Characteristic	Total Enrolled and Tested (*n* = 1248)	RSV (*n* = 32/1248) 2·6%	HMPV (*n* = 33/1248) 2·6%
Age (years), median (25%, 75%)	51·1 (35·3, 65·5)	60·8 (44·8, 68·9)	61 (49·3, 67·3)
Age group, *n* (%)			
18–49 years	604 (48·4)	12 (37·5)	10 (30·3)
≥50 years	644 (51·6)	20 (62·5)	23 (69·7)
Duration of symptoms in days, median (25%, 75%)	3 (1, 5)	4 (2, 6)	3 (2, 4)
Sex, *n* (%)			
Male	513 (41·1)	11 (34·4)	10 (30·3)
Female	735 (58·9)	21 (65·6)	23 (69·7)
Race/Ethnicity, *n* (%)			
White	827 (66·3)	25 (78·1)	23 (69·7)
Black	386 (30·9)	7 (21·9)	10 (30·3)
Living situation, *n* (%)			
Lives alone	231 (18·5)	11 (34·4)	6 (18·2)
Lives with family	961 (77)	20 (62·5)	27 (81·8)
In nursing facility	43 (3·5)	1 (3·1)	0 (0)
Chronic illnesses, *n* (%)	1013 (81·2)	28 (87·5)	29 (87·9)
Cardiovascular disease	427 (34·2)	15 (46·9)	14 (42·4)
Pulmonary disease	671 (53·8)	22 (68·8)	21 (63·6)
Diabetes mellitus	302 (24·2)	7 (21·9)	6 (18·2)
Immunodeficiency*	680 (54·5)	19 (59·4)	24 (72·7)
Exposure to tobacco smoke**, *n* (%)	579 (46·4)	16 (50)	11 (33·3)

HMPV, human metapneumovirus; IQR, interquartile range; RSV, respiratory syncytial virus.

* Transplant, cancer, splenectomy, HIV/AIDS, steroid use, chemotherapy, haemoglobinopathy, immunosuppression.

** Smoked or had significant environmental tobacco exposure within the last 6 months.

### Clinical presentation of specific viral infection

Of the 1248 study patients, 32 (2·6%) had RSV identified, including 14 of 711 (2%) ED patients and 24 of 835 (2·9%) hospitalized patients. A total of 33 (2·6%) patients had HMPV identified, including 1·8% of ED patients and 3·2% of hospitalized patients. No patient had both of these viruses or influenza co-infection identified. Gender, race, and living situation (independent or with family) were not associated with specific viral infections. Most patients (57/65, 87·7%) infected with one of the study viruses had at least one medical co-morbidity (Table 1). Overall median duration of symptoms before presentation, inpatient length of stay, admission to and length of stay in the intensive care unit, need for mechanical ventilation, and death were not different between those hospitalized with either of the tested viruses (Table 2). Demographic characteristics and clinical signs and symptoms of patients infected with RSV and HMPV were similar (Tables1 and 2). One patient with RSV died before hospital discharge. This patient was a 68-year-old Caucasian woman with cancer who was receiving chronic corticosteroid therapy.

**Table 2:** Symptoms at enrollment and clinical outcomes for patients with RSV and HMPV

Characteristic	RSV infection (*n* = 32)	HMPV infection (*n* = 33)	*P* value
Duration of symptoms in days, median (25%, 75%)	4 (2, 6)	3 (2, 4)	0·670
Symptoms/Signs, *n* (%)			
Congestion/Rhinorrhea	25 (78·1)	24 (72·7)	0·614
Sore throat	19 (59·4)	13 (39·4)	0·107
Cough	29 (90·6)	32 (97)	0·287
Dyspnea	30 (93·8)	32 (97)	0·536
Wheezing	29 (90·6)	26 (78·8)	0·186
Earache	11 (34·4)	6 (18·2)	0·137
Fever	19 (59·4)	23 (69·7)	0·461
GI symptoms	13 (40·6)	18 (54·6)	0·261
Decreased appetite	23 (71·9)	25 (75·8)	0·722
Myalgias	17 (53·1)	19 (57·6)	0·718
Headache	21 (65·6)	19 (57·6)	0·505
Fatigue	31 (96·9)	27 (81·8)	0·050
Altered mental status	7 (21·9)	9 (27·3)	0·614
Clinical outcomes among those hospitalized *N*	*N* = 24	*N* = 27	
Length of stay in days, median (25%, 75%)	4 (2, 5)	3 (2, 4)	0·877
ICU admission, *n* (%)	4 (16·7)	2 (7·4)	0·306
Length of stay in ICU in days, median (25%, 75%)	3 (2·5, 5·0)	4·5 (1·0, 8·0)	0·489
Need for mechanical ventilation, *n* (%)	1 (4·2)	0 (0)	0·284
Death, *n* (%)	1 (4·2)	0 (0)	0·284

### Viral-specific hospitalization rates

**Figure 2:**
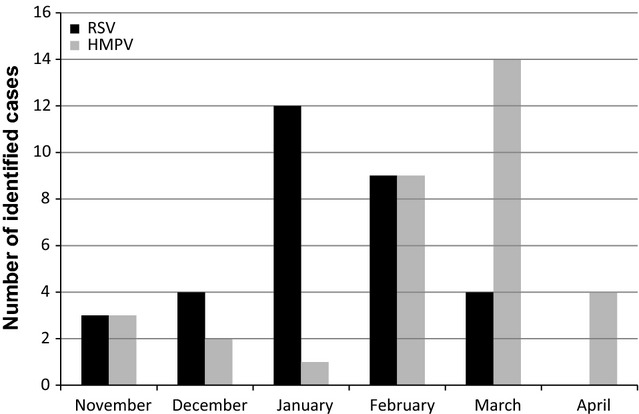
Number of adults enrolled with ARI-associated ED visit or hospitalization with RSV or HMPV identified by month, May 2009 through April 2010.

RSV circulated from November 2009 through March 2010 and HMPV circulated from November 2009 through April 2010 (Figure 2). RSV peaked in January, while HMPV peaked in March. Over the 12-month period studied, ED visits in Middle Tennessee per 10 000 adults aged 18–49 were 13·18 (95% confidence interval (CI) [6·72, 25·27]) for RSV and 10·13 (95% CI [4·67, 21·61]) for HMPV. ED rates per 10000 adults 50 years and older were 19·48 (95% CI [9, 40·8]) for RSV and 34 (95% CI [16·61, 67·74]) for HMPV. Hospitalization rates per 10000 adults aged 18–49 years were 2·11 (95% CI [1·04, 4·23]) for RSV and 1·81 (95% CI [0·83, 3·82]) for HMPV. Hospitalization rates per 10,000 adults 50 years and older were 11·24 (95% CI [7·07, 17·69]) for RSV and 14·21 (95% CI [9·37, 21·3]) for HMPV (Table 3).

**Table 3:** Rates per 10 000 of ED visits and hospitalizations in patients aged 18–49 years, 50–64 years, and 65 years and older

	RSV Infection		HMPV Infection	
Age Groups	ED *N* = 14	Inpatient* *N* = 24	ED *N* = 13	Inpatient* *N* = 27
18–49	13·18 (6·7, 25·3)	2·11 (1, 4·2)	10·13 (4·7, 21·6)	1·81 (0·8, 3·8)
50–64	12·76 (4·4, 35·4)	6·71 (3·3, 13·4)	41·29 (17·8, 91·8)	9·76 (5·4, 17·3)
65+	33·96 (11·7, 90·8)	18·96 (10·4, 34)	23·12 (6·3, 78)	21·41 (12·1, 37·3)
Total 50+	19·48 (9, 40·8)	11·24 (7·1, 17·7)	34 (16·6, 67·7)	14·21 (12·1, 37·3)
Total 18+	15·44 (9·3, 25·4)	5·5 (3·7, 8·1)	14·64 (8·6, 24·7)	6·28 (4·3, 9)

* Patients originally evaluated in the ED and then admitted were counted in both the ED and the Inpatient columns because they used resources in both healthcare settings.

## Discussion

In adults 50 years and older, we found substantial rates of hospitalization associated with both RSV and HMPV detection, about 10–20 per 10 000, which is very similar to annual influenza-associated hospitalization rates reported for this age group.^1^,^6^–^11^ ED visit rates for RSV and HMPV in older adults were approximately 2 times higher than hospitalization rates. In adults aged 18–49 years, rates of hospitalization associated with RSV and HMPV were about 2 per 10 000 and ED rates were 5–6 times higher.

There have been few previous studies of the rates of hospitalizations and ED visits due to RSV in adults and almost no prior estimates for HMPV. Two studies estimated RSV hospitalization rates based on models of seasonal trends in cardio-respiratory hospitalizations and influenza and RSV surveillance data. Muloolly, *et al.,*^12^ estimated annual RSV hospitalization rates among adults without identified high-risk conditions to be 0·2, 0·9, and 10·6 per 10 000 persons aged 18–49, 50–64, and ≥65 years. Among adults with high-risk conditions, rates were estimated to be 1·2 (95% CI [−0·9, 3·4]), 7·2 (95% CI [1·9, 12·4]), and 44·0 (95% CI [31·0, 57·0]) per 10 000 persons 18–49, 50–64, and ≥65 years, respectively. The rates we determined for all adults were similar to their estimates for those with high-risk conditions in adults aged <65 years, and in between their estimates for low and high risk for those aged 50–64 years. Zhou, *et al*.,^11^ using a model similar to that of Muloolly, estimated average annual RSV hospitalization rates over 15 consecutive years to be 1·28 and 8·61 per 10 000 adults aged 50–64 and 65 years and older, respectively. These rates are comparable to those estimated by Muloolly for low risk persons, but considerably lower than our rates for all adults.

The RSV hospitalization rate of 11/10 000 for adults 50 years and older is similar to that of 15/10 000 for the same age group, reported in our prior three-year study, which also used direct measurement of RSV.^1^ Estimated RSV hospitalization rates in both modeling studies were considerably lower. RSV is difficult to diagnosis in adults because antigen testing sensitivity is so poor.^13^ Therefore, estimation of disease due to RSV is likely low if based on historical clinical laboratory testing. Our RSV hospitalization rates for adults 50 years and older were similar to influenza hospitalization rates during the same period. Falsey, *et al*.,^14^ reported that a similar proportions of adult patients hospitalized with acute respiratory illness (ARI) had influenza and RSV detected when using RT-PCR to diagnose RSV; however, they did not estimate hospitalization rates.

Little is known about HMPV in adults. The virus was first described in 2001 and was originally thought to cause respiratory infections only in children.^15^ Subsequently, significant disease in adults with mortality as high as 50% in frail elderly residents has been documented.^16^ We were able to show that in adults, hospitalization rates increased significantly with age. This new knowledge provides evidence that HMPV causes significant morbidity at the extremes of age.

Because this study was performed during the H1N1 influenza pandemic year, comparison of rates of ED visits and hospitalizations due to pandemic influenza can be directly compared to RSV and HMPV ED visit and hospitalization rates. Using similar methods, Jules, *et al*., 8 estimated 14·3 hospitalizations per 10 000 adults aged ≥50 years for influenza during May 2009 through March 2010, and Self, *et al*., 17 estimated 36 ED visits per 10 000 (95% CI [10·5, 77·7]) adults aged ≥50 years during the same time period. Both studies found rates that approximate the rates we estimated for both RSV- and HMPV-associated illness.

Our study has several limitations. Our sample size was small, making it difficult to draw comparisons of the clinical presentations associated with each virus. In addition, because this study took place during the novel influenza A H1N1 pandemic and was only 1 year, rates may not be representative of all years. However, annual hospitalization rates for adults 50 years and older were similar to those we had reported for the prior 3 years for RSV. Furthermore, this study only included the middle Tennessee region and relied on the assumption that the percent of infections in persons we enrolled would be similar to percent in persons with acute respiratory illness ED and hospital discharge diagnoses. Despite these limitations, studies such as this that use direct patient testing to estimate rates are needed to help determine the validity of modeling studies.

RSV and HMPV are associated with a significant number of ED visits and hospitalizations in adults ≥50 years of age, especially in those ≥65 years of age. Previous studies have shown that older adults with influenza do not always display typical symptoms, making the sensitivity of clinical diagnosis low.^18^,^19^ As the clinical presentations of RSV and HMPV are similar to that of influenza, awareness and clinical suspicion, in addition to the use of sensitive molecular diagnostic methods, are needed to detect and distinguish these infections. In addition, rates of hospitalization associated with RSV and HMPV were higher than those due to influenza in adults ≥65 years of age that we reported in our previous 3-year study. This is likely due in part to high vaccination of the older population in the United States. To date, efforts to limit the deleterious effects of RSV and HMPV infection have involved mainly infection control measures, including good hand hygiene practices. With such a high burden of illness, there is significant need for vaccines and treatment for both RSV and HMPV.

In summary, during 2009–2010, when pandemic influenza was circulating, hospitalization rates for RSV and HMPV in those ≥50 years of age were similar to those reported for influenza, about 10 per 10 000. ED rates were approximately 2 times higher than hospitalization rates. For those aged 18–49 years, hospitalization rates were about 2 per 10 000 with ED rates five to six times higher.
